# Interprofessional collaboration between hospital-based palliative care teams and general healthcare workers: A realist review protocol

**DOI:** 10.1371/journal.pone.0310709

**Published:** 2024-11-11

**Authors:** Louana Moons, Fouke Ombelet, Mieke Deschodt, Maaike L. De Roo, Eva Oldenburger, Inge Bossuyt, Peter Pype

**Affiliations:** 1 Department of Public Health and Primary Care, Gerontology and Geriatrics, KU Leuven, Leuven, Belgium; 2 Department of Palliative Medicine, University Hospitals Leuven, Leuven, Belgium; 3 Department of Neurology, University Hospitals Leuven, Leuven, Belgium; 4 Laboratory of Neurobiology, Center for Brain & Disease Research, VIB, Leuven, Belgium; 5 Department of Neurosciences, Leuven Brain Institute (LBI), KU Leuven, Leuven, Belgium; 6 Competence Center of Nursing, University Hospitals Leuven, Leuven, Belgium; 7 Department of Geriatrics, University Hospitals Leuven, Leuven, Belgium; 8 Department of Radiation Oncology, University Hospitals Leuven, Leuven, Belgium; 9 Department of Public Health and Primary Care, Unit Interprofessional Collaboration in Education, Research, and Practice, Faculty of Medicine and Health Sciences, Ghent University, Ghent, Belgium; Universiteit Antwerpen, BELGIUM

## Abstract

**Background:**

Palliative care, vital for patients with advanced, life-limiting or life-threatening illnesses, faces an increasing global demand due to aging populations and rising non-communicable diseases. Specialized palliative care teams (PCTs) within hospitals significantly impact patient outcomes, which requires effective interprofessional collaboration with general healthcare workers. Therefore, We will conduct a realist review to explore the contextual factors, mechanisms and outcomes related to the interprofessional collaboration between PCTs and general healthcare workers.

**Methods:**

Grounded in the third generation Cultural-Historical Activity Theory, this review will follow 5-step iterative process. First, a preliminary literature search will define the review scope. Second, based on the preliminary searches an initial program theory will be developed. Third, systematic searches across PubMed, Embase, CINHAL, Web of Science, and Scopus will be caried out. Fourth, data extraction of included studies will be conducted. Simultaneously, relevance and rigour of individual studies will be evaluated. Lastly, data analysis and synthesis will be conducted in which identified individual Context-Mechanism-Outcome (CMO) configurations will be combined in chains of inference through which hypotheses can be formulated. In summary, this realist review will refine an initially developed program theory, producing a framework elucidating how interprofessional collaboration works between PCTs and general healthcare workers.

**Discussion:**

This review aims to provide crucial insights into interprofessional collaboration between PCTs and general healthcare workers, informing optimized palliative care delivery in acute care hospitals for diverse stakeholders.

## Introduction

According to the WHO, palliative care is defined as “the approach that improves quality of life of patients and their families who are facing problems associated with life-threatening illness. Palliative care prevents and relieves suffering through early identification, impeccable assessment and treatment of all problems, whether physical, psychosocial, or spiritual” [1]. Patients with an advanced, life-limiting disease often face a period of physical, psychological and cognitive decline before death ensues [2]. Given the rapid growth and ageing of the global population, it is projected that the worldwide death toll will surge over the coming decades, escalating from 67 million in 2022 to an anticipated 92 million by 2050 [3]. This demographic shift highlights an increasing demand for palliative care, which was required for 45.3% of all deaths worldwide in 2017 [4]. Additionally, a range of 10%-78% of deaths occur in hospital, depending on location, population, and study type [5–10]. Therefore, we need to ensure good quality palliative care within the hospital setting.

Several international and national organizations provide guidelines and recommendations for palliative care delivery in the hospital setting. For example in Europe, guidelines are developed by the European Association for Palliative Care (EAPC) which recommends two specialized palliative care services for every 100.000 adult inhabitants, consisting of one home care team and one hospital team [11]. Overall, the use of specialized, hospital-based palliative care teams (PCTs) plays a central role in the organization of palliative care worldwide [12–16]. Involvement of these PCTs is associated with improved outcomes in both patients with advanced illnesses (e.g. quality of life, symptom burden, spiritual wellbeing) as well as family members and general healthcare workers (HCWs) (e.g. satisfaction with care provided) [17–24]. In addition, the initiation of palliative care consultations at the early stages of hospital admission is linked to a lower cost of hospital stay [20].

A PCT generally aims to provide support and advice concerning palliative care to general HCWs and tries to bring palliative care principles into the acute care hospital wards, rather than to take on the primary responsibility for the patient or to take over patient care [25–27]. Hence, general HCWs remain responsible for the care provided to hospitalized palliative patients [28]. This highlights the importance of adequate interprofessional collaboration (IPC) between PCTs and general HCWs within the hospital setting. Multiple definitions of IPC exist, yet the predominant ones consistently delineate common themes [29–33]. We define IPC as the collaborative practice between multiple HCWs from different professional backgrounds in which shared accountability, interdependence, and clarity of roles are of importance to deliver the highest quality of care to patients, their families, and carers [29–33].

In general, an effective IPC practice could lead to improved access to healthcare, efficient use of resources, and increased job satisfaction among HCWs [34]. However, when looking at the collaborative practices among PCTs and general HCWs it is still unclear how, why and under which circumstances this type of IPC leads to improved outcomes. Reviews found that PCTs enhance collaboration between the referring general HCW and the palliative care specialist in which collaboration is fostered by recognizing the expertise of the other. IPC between PCT members and general HCWs is facilitated through effective communication, complementary and clear roles, opportunities for education, shared problem-solving, and continuous support [25, 28]. However, little consistency exists in how palliative care models are developed and how their success is evaluated [28]. These evidence gaps may be due to a limited understanding of the operational procedures currently employed by PCTs, particularly in the domain of IPC with general HCWs. In order to optimize the quality of palliative care within the hospital setting we need a more profound understanding of these collaboration processes between PCTs and general healthcare and its mediating factors.

We strive to gain a better understanding about how, for whom and under which conditions IPC between PCTs and general HCWs of acute care hospital wards contributes to the quality of care for everyone involved (e.g. patient’s quality of life, care team satisfaction, cost efficiency) [35–37]. Hence, this realist review aims 1) to identify different components and conditions leading to a successful IPC between PCTs and general HCWs, 2) to determine for whom IPC is successful and for whom it is not, and 3) to pinpoint current outcomes that are used to evaluate the impact of these IPC processes.

## Methods

### Realist review

A realist review is an iterative, theory-driven technique helping to make sense of heterogeneous evidence about complex interventions applied in diverse contexts [38, 39]. The realist philosophy behind this type of review agrees that a real world exists, but that it is perceived as, interpreted as, and responded to through human senses, brains, language and culture [39, 40]. Realism involves identifying causal mechanisms and exploring how these work in particular contexts generating the observed outcomes [39, 41]. Mechanisms are defined as the underlying entities, processes, or structures which operate in particular contexts to generate outcomes of interests [39] They can be seen as the hidden components of an intervention that cause change [38]. Context refers to broad social or geographical features, factors influencing program implementation, participant composition, population profiles in intervention locations, and conditions influencing subjects’ choices [40]. Outcomes can be seen as the observed changes that result from the delivery of an intervention within a certain context using certain mechanisms [42]. The realist approach to literature research is fundamentally concerned with program theory development which is able to describe the theoretical relationship between contexts, mechanisms and outcomes. These program theories are then tested against empirical evidence and refined to better explain how a complex intervention works, for whom, and in which conditions [42].

**Fig 1:**
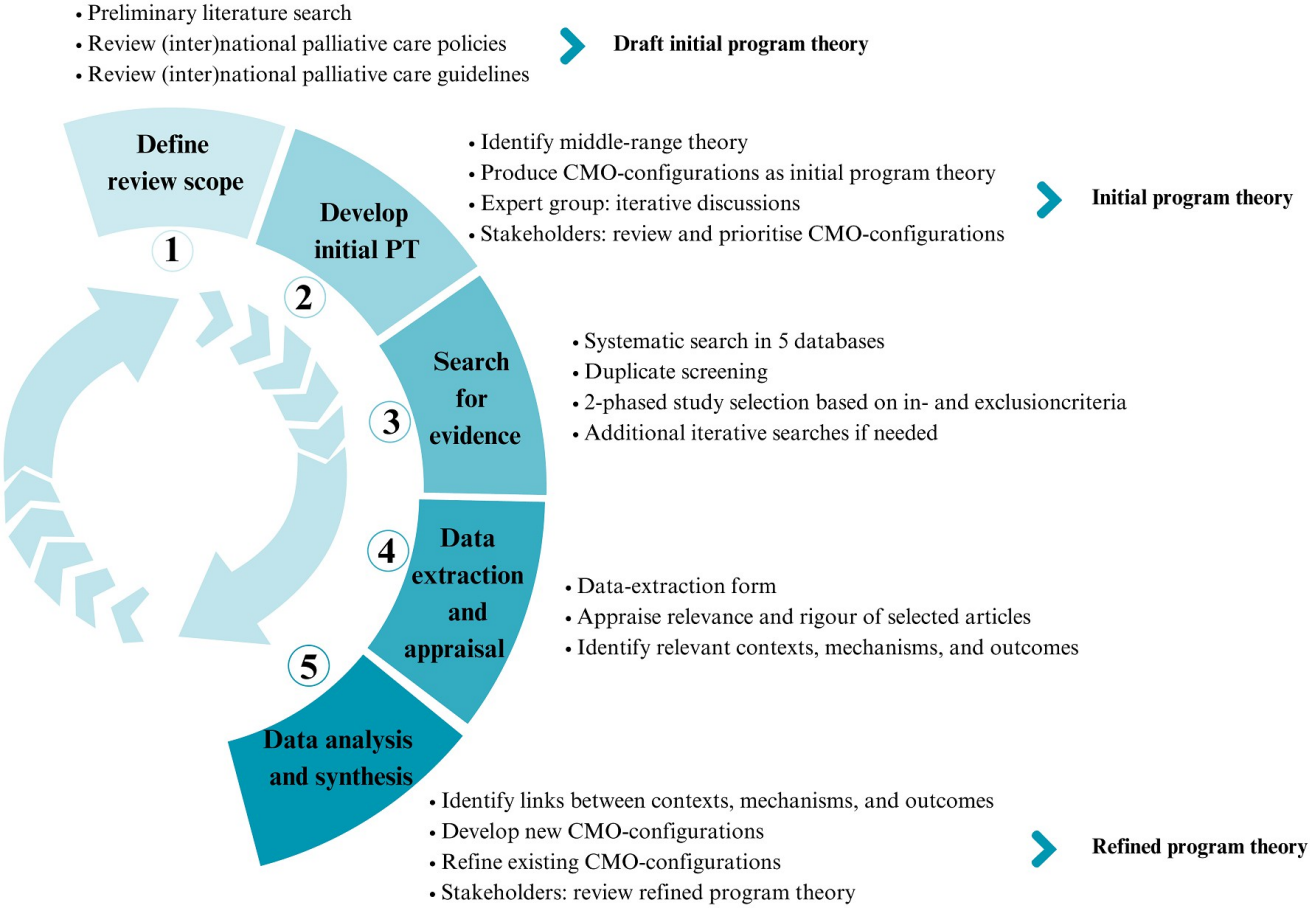
Summary of the review design.

This review protocol and its research questions were developed by our research team, which includes experts in palliative care, implementation research and IPC. The realist review will be guided by 5 steps, as described by Pawson and colleagues): 1) define the review scope, 2) develop an initial program theory, 3) search for evidence, 4) data-extraction and appraisal 5) data analysis and synthesis [42]. Although the different steps are presented sequentially, the realist review process is iterative, which may lead to overlap or parallel progression of these steps as the review progresses. Fig 1 offers a summary of the review design.

### Step 1: Define the review scope

A realist review starts by defining the review’s scope and formulating initial program theories that can serve as a framework for synthesizing evidence [42]. Therefore, we will conduct a preliminary search of the existing literature concerning IPC within the palliative care setting in hospitals to help determine the scope of our review and to provide us with a starting point for the development of our initial program theory.

The realist review questions to guide our preliminary literature search to define our review scope are:

1. Through which mechanisms does IPC between PCTs and general HCWs work?
2. Which aspects of IPC processes between PCTs and general HCWs contribute to the quality of care for everyone involved?
3. In which conditions does IPC between PCTs and general HCWs contributes to the quality of care for everyone involved?
4. By whom is the IPC between PCTs and general HCWs perceived to be successful?
5. What are the measurable outcomes influenced by IPC between PCTs and general HCWs on patient, family, HCWs, and hospital level?

To identify relevant articles we will do preliminary searches in PubMed using search terms such as “palliative care”, “palliative care teams”, and “interprofessional collaboration”. If possible, Mesh-terms and Boolean operators will be used in these preliminary searches. We will also carry out focused backward citation searching for articles of which we suspect their references can contribute to the preliminary informational scope of our search. Lastly, we will review national and international palliative care policy documents and guidelines concerning IPC.

### Step 2: Develop an initial program theory

The preliminary search described in step 1 will result in an initial program theory highlighting key mechanisms, contextual factors, and relevant outcomes related to IPC between PCTs and general HCWs from acute care hospital wards. A program theory is defined as a theory that provides a framework about how an intervention is expected to work [40]. Within our realist review we aim to develop a program theory that includes descriptions of contexts; *in which conditions does the intervention work*?, mechanisms; *how does the intervention work*?, and outcomes: *what are the observed outcomes of the intervention*?. Overall, the aim is to produce a program theory consisting of context, mechanism, and outcome (CMO-) configurations which will highlight not only whether the intervention works but also how it works, for whom and in what circumstances.

**Fig 2:**
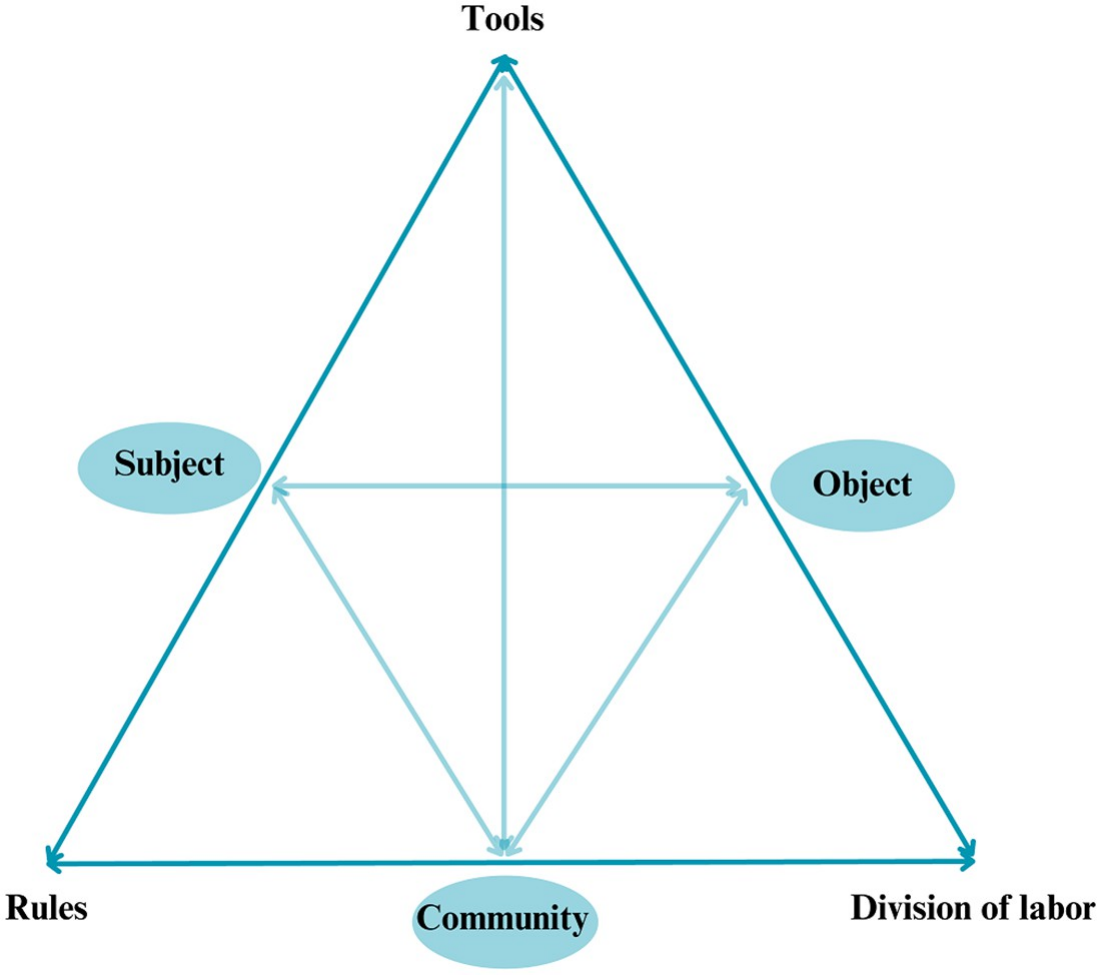
The activity system as a basic unit of analysis in CHAT. Source: Engeström (1987, p. 213) © Yrjö Engeström. Adapted by permission of Yrjö Engeström. Permission to reuse must be obtained from the rightsholder.

Based on the recommendation that an initial program theory should ideally be grounded in a middle-range theory, we have chosen the third generation Cultural-Historical Activity Theory (CHAT) as a suitable middle-range theory for this review [38]. Healthcare environments, especially those involving palliative care, are inherently complex. CHAT is a socio-cultural and socio-material theory and was developed for analyzing and better understanding practices in complex learning environments in relation to their cultural and historical contexts [43–45]. The basic unit of analysis in CHAT is a multi-voiced and multi-layered *activity system* consisting of six core components: the subject, the object, tools, community, rules, and division of labor [44, 46]. Fig 2 shows this basic unit of analysis in CHAT [47].

In CHAT’s third generation, this consideration expands to two or more interacting activity systems. This is particularly relevant to our realist review as IPC involves various stakeholders, such as PCTs, general healthcare workers, and patients engaging in different yet interconnected activity systems. For example, in a first activity system the object might be to provide efficient palliative care in the acute care hospital setting and the subjects within this activity system are the PCT-members. The activity, IPC, is mediated by tools (e.g. the use of a shared electronic health record), and the subjects, PCT-members, interact with the community (e.g. general HCWs), rules (e.g. palliative care guidelines), and division of labour (e.g. specific PCT responsibilities). Furthermore, the general HCWs undergoing the activity (IPC) can also be conceptualized as a separate, but interacting activity system. Therefore, we believe the third generation of CHAT will be a useful theoretical framework to investigate how, for whom, and under which circumstances current IPC between PCTs and general HCWs of acute care hospitals leads to an impact.

We will develop an initial program theory through preliminary scoping the literature, as well as through discussion rounds with the expert of this realist review. In addition, we will involve stakeholders (IB, EO) that are familiar with the research topic to review and prioritize the main CMO-configurations within our initial program theory. This initial program theory will then serve as the starting point for our data analysis and synthesis in which we aim to refine the program theory based on the empirical evidence that was found through the search for evidence, described in step 3.

### Step 3: Search for evidence

In this step suitable articles will be identified to test and refine the initial program theory. We will search the following electronic databases: PubMed, Embase, CINHAL, Web of Science (WoS), and Scopus. We have already developed a search strategy in close collaboration with experts from the KU Leuven Libraries, consisting out of three syntaxes: “Hospital”, “Palliative Care” and “Patient Care Team”. Our final search strategy for PubMed can be found in (S1 File). This search strategy will be adapted according to the specific requirements of all five databases. We will limit the search to articles published in the last ten years. By doing this, we aim to focus on the current organization and processes regarding IPC between PCTs and general HCWs. After running the search in all databases we will import the metadata into Endnote^®^, as a reference management program, for duplicate screening. An 11-step deduplication process will be followed based on the recommended blogpost of Jane Falconer (2018) by KU Leuven Libraries [48]. This duplicate screening will result in a final set of articles which will be imported into Rayyan, a data management software program, which will be used for study selection.

Study selection will be carried out in two phases by two independent researchers (FO and LM). First screening will be based on title and abstract. The second screening will be based on full texts resulting in a final set of articles included in the realist review. A preliminary set of in- and exclusion criteria has been developed mainly focusing on study design, language, setting and population. Articles with a qualitative, quantitative, or mixed-method design will be included when written in English or Dutch, carried out in an acute care hospital setting, and involve adult patients who receive palliative care through IPC between PCTs and general HCWs. During title and abstract screening, several discussion rounds between the researchers (FO and LM) will be held to discuss and refine these in- and exclusion criteria in order to focus the review throughout the screening procedure. When no consensus is found during these discussion rounds, a third researcher (MD) will be consulted. Once the in- and exclusion criteria are final, earlier screened articles will be rescreened on title and abstract in order to make sure all relevant articles are included based on our final set of in- and exclusion criteria. During both phases of study selection, arising conflicts will be discussed between the two researchers until consensus is reached. If, at any time during the data selection process, conflicts cannot be resolved a third researcher will be involved to obtain consensus. We will not consider grey literature in this realist review, as there is a considerate amount of available peer-reviewed papers which will already provide a sufficient breadth of study designs, ensuring a comprehensive coverage of our topic.

According to the RAMESES training materials, new insights might arise during data-analysis which means additional iterative searches might be needed as the review progresses [40]. If that would be the case, we will purposively search for evidence to support or refute these emerging new pathways. It is thus dependent on the exhaustiveness of the data we will reach during our data appraisal and analysis stage if additional searches will be needed to be carried out.

### Step 4: Data-extraction and appraisal

In this step we will extract all relevant data into a data extraction form. This form will be piloted on 10 studies and refined as necessary. Data will be extracted by one researcher (LM) and validated by another researcher (FO). From each article, we will extract title, aim, design, participants, setting, intervention, outcomes, limitations and conclusion. During this data-extraction process key passages related to any mechanism, contextual factor or outcome related to IPC will be highlighted manually by using different colors to code different passages. Simultaneously, we will critically appraise all included articles for their fitness for purpose based on two important concepts: relevance and rigour. Relevance ranges from ‘low’, ‘medium’, to ‘high’ and is based on whether and to what extent the article contributes to the theory refining process (40, 49). Rigour of the relevant data is assessed through the credibility and trustworthiness of the methods used. Articles will receive a score of either ‘low’ or ‘high’ rigour [40, 49].

### Step 5: Data analysis and synthesis

For the data analysis, we will examine all manually highlighted passages from step 4 for insights into intervention mechanisms, contextual factors and outcomes related to IPC. Iteratively, the data synthesis will be carried out to refine our understanding in how the intervention works, for whom it works and for whom it does, under what circumstances and/or which settings, and why it is expected to work. As little guidance is provided on how to approach this step in a practical way, we will follow a 5-step approach described by Rycroft-Malone and colleagues [41]:

1. Organization of extracted data into evidence tables.
2. Theming by individual reviewers.
3. Comparison of reviewers’ themes for a specific article and formulation of chains of inference from the identified themes.
4. Linking of the chains of inference, and tracking and linking of articles.
5. Hypothesis formulation.

In a realist review, the fundamental analytical task is to identify and align evidence that illustrates the specific mechanisms leading to particular outcomes within certain contexts. As a first step, we will organize our data extraction tables based on their relevance and rigour. Secondly, full text articles will be analyzed to identify individual CMO configurations in accordance to the research question. Articles with the highest relevance and rigour will be examined independently by two researchers (LM, FO). Multiple meetings between both researchers will be held to resolve any discrepancies and to align their analytical approach. If consensus within these primary articles is achieved, the remaining articles will be analyzed by one researcher (LM) and validated by another (FO, MD or PP). Third, we will try to identify and formulate chains of inference which are the connections across articles based on the identified themes. To do so, timely and repetitive meetings will be held with the complete research team. Fourth, we will link the identified chains of inferences to different hypotheses in order to test these. Thus, identified themes will be linked to chains of inferences and these chains of inferences will then be linked to a hypothesis.

Simultaneously with data analysis, we will start with data synthesis. The basic task within this process is to refine the initial program theory in order to achieve a finetuned understanding of how IPC between PCTs and general HCWs works. In this last phase of the review, we will iteratively test and refine our program theory based on explanations we found through the empirical findings within our included studies. We will tap into stakeholders and experts as well as reflection and discussion among the review team. The end goal of this realist review process is the refinement of our initial program theory and the production of a new framework explaining how IPC between PCTs and general HCWs is intended to work, for whom and in which circumstances.

We have filled in the PRISMA-P Checklist to enhance the methodological oversight of this review (S2 File). In addition, we will report our findings according to the Realist And Meta-narrative Evidence Synthesis: Evolving Standards (RAMESES) quality and publication standards (2014) [39, 50]. As this realist review will collect previously published data and will not involve human or animal participants, no ethical approval is necessary to be obtained. All data extraction forms will be made available as part of the publication of the realist review.

## Discussion

Performing a realist review involves several practical and operational considerations to take into account. To start, realist reviews often deal with heterogeneous evidence, including diverse study designs and methodologies. However, we believe to have developed a strategy that will help make sense of the heterogeneity within our review. First, by using CHAT as a middle-range theory we will be able to maintain focused on different processes and mechanisms that will reveal during data analysis. Second, both the RAMESES reporting guidelines and training materials will provide us guidance to work systematically and collect only relevant information in relation to our research question. At last, by involving relevant stakeholders in the theory development we will be able to focus on processes, contextual factors and outcomes which are perceived the most relevant for current palliative care practice. A second consideration to make is about the iterative nature of a realist review and the extensive program theory refinement process. These are considered as strengths of the realist methodology, however they do present the challenge of it might being a time consuming process. Finally, a realist review deals with complex interventions and their interactions within contextual factors. Managing this complexity and developing a refined program theory that explains certain outcomes might be challenging. Therefore, we will continue to foster an open communication within the research team to address arising problems, challenges, and complexities.

The conduct of this realist review will allow us to explore the relationships between contexts, mechanisms and outcomes related to the IPC between PCTs and general HCWs. Overall, we will gain a better understanding of ‘what works, for whom, under which conditions and why’. In summary, this review has the potential to impact the healthcare landscape by providing novel insights into this topic, which will allow health professionals, care coordinators, hospital managers and policy makers to optimize current palliative care delivery in the acute care hospital setting.

## Supporting information

S1 File Search strategy. (PDF)

S2 File PRISMA-P checklist. (PDF)
