# Supplementary material for: Interprofessional collaboration between hospital-based palliative care teams and general healthcare workers: A realist review protocol
Source: PLoS One. 2024 Nov 11;19(11):e0310709. doi: 10.1371/journal.pone.0310709 (PMC11554213; doi:10.1371/journal.pone.0310709)
Supplement: S1 File — (PDF) [file pone.0310709.s001.pdf]

<u>Supporting information file 1:</u> Final search strategy PubMed

"Hospitals"[MeSH Terms:noexp] OR "hospital"[Title/Abstract] OR "Hospital Medicine"[MeSH Terms] OR "Hospitalization"[MeSH Terms:noexp] OR "Hospital Departments"[MeSH Terms:noexp] OR "hospitals, general"[MeSH Terms] OR "oncology service, hospital"[MeSH Terms] OR "cardiology service, hospital"[MeSH Terms] OR "oncology ward*"[Title/Abstract] OR "oncological ward*"[Title/Abstract] OR "oncologic ward*"[Title/Abstract] OR "oncology department*"[Title/Abstract] OR "oncological department*"[Title/Abstract] OR "oncologic department*"[Title/Abstract] OR "oncology unit*"[Title/Abstract] OR "oncological unit*"[Title/Abstract] OR "oncologic unit*"[Title/Abstract] OR "geriatric ward*"[Title/Abstract] OR "geriatric department*"[Title/Abstract] OR "geriatric unit*"[Title/Abstract] OR "pneumology ward*"[Title/Abstract] OR "pneumology department*"[Title/Abstract] OR "pneumology unit*"[Title/Abstract] OR "pulmonology ward*"[Title/Abstract] OR "pulmonology department*"[Title/Abstract] OR "pulmonology unit*"[Title/Abstract] OR "cardiology ward*"[Title/Abstract] OR "cardiology department*"[Title/Abstract] OR "cardiology unit*"[Title/Abstract] OR "cardiological ward*"[Title/Abstract] OR "cardiological department*"[Title/Abstract] OR "cardiological unit*"[Title/Abstract] OR "cancer ward*"[Title/Abstract] OR "cancer department*"[Title/Abstract] OR "cancer unit*"[Title/Abstract]*

AND

"Palliative Care"[MeSH Terms] OR "palliat"[Title/Abstract] OR "Terminal Care"[MeSH Terms] OR "Terminal Care"[Title/Abstract] OR "end of life care*"[Title/Abstract] OR "eol care*"[Title/Abstract] OR "symptomatic treatment*"[Title/Abstract] OR "euthanasia*"[Title/Abstract] OR "resuscitation order*"[Title/Abstract] OR "resuscitation decision*"[Title/Abstract] OR "assisted suicide*"[Title/Abstract] OR "assisted death*"[Title/Abstract] OR "Advance Care Planning"[MeSH Terms:noexp] OR "Advance Care Planning"[Title/Abstract] OR "advance health care planning"[Title/Abstract] OR "advance medical planning"[Title/Abstract] OR "Hospice and Palliative Care Nursing"[MeSH Terms] OR "Hospices"[MeSH Terms] OR "hospice*"[Title/Abstract] OR "bereavement care"[Title/Abstract] OR "bereavement support"[Title/Abstract] OR "grief support"[Title/Abstract] OR "Palliative Medicine"[MeSH Terms] OR "Terminally Ill"[MeSH Terms] OR "terminally ill*"[Title/Abstract] OR "terminal ill*"[Title/Abstract] OR "terminal patient"[Title/Abstract:~2] OR "terminal patients"[Title/Abstract:~2] OR "terminally patient"[Title/Abstract:~2] OR "terminally patients"[Title/Abstract:~2] OR "dying patient*"[Title/Abstract] OR "Withholding Treatment"[MeSH Terms] OR "withholding treatment*"[Title/Abstract] OR "withdrawing treatment*"[Title/Abstract] OR "treatment withdrawal*"[Title/Abstract] OR "withholding care"[Title/Abstract] OR "withdrawing care"[Title/Abstract] OR "care withdrawal"[Title/Abstract] OR "treatment cessation"[Title/Abstract] OR "cessation of treatment"[Title/Abstract] OR "cessation of care"[Title/Abstract]*

AND

"Patient Care Team"[MeSH Terms] OR "health care team"[Title/Abstract] OR "healthcare team*"[Title/Abstract] OR "patient care team"[Title/Abstract:~2] OR "patient care teams"[Title/Abstract:~2] OR "medical care team"[Title/Abstract:~2] OR "medical care teams"[Title/Abstract:~2] OR "collaborative team"[Title/Abstract:~2] OR "collaborative teams"[Title/Abstract:~2] OR "interdisciplinary team"[Title/Abstract:~2] OR "interdisciplinary teams"[Title/Abstract:~2] OR "inter-disciplinary team"[Title/Abstract:~2] OR "inter-disciplinary teams"[Title/Abstract:~2] OR "multidisciplinary team"[Title/Abstract:~2] OR "multidisciplinary teams"[Title/Abstract:~2] OR "multi-disciplinary team"[Title/Abstract:~2] OR "multi-disciplinary teams"[Title/Abstract:~2] OR "transdisciplinary team"[Title/Abstract:~2] OR "transdisciplinary teams"[Title/Abstract:~2] OR "trans-disciplinary*

team"[Title/Abstract:~2] OR "trans-disciplinary teams"[Title/Abstract:~2] OR "decision making, shared"[MeSH Terms] OR "SDM"[Title/Abstract] OR "shared decision"[Title/Abstract] OR "sharing decision*"[Title/Abstract] OR "shared learning"[Title/Abstract] OR "collaborative learning"[Title/Abstract] OR "Cooperative Behavior"[MeSH Terms] OR "cooperative behavior*"[Title/Abstract] OR "cooperative behaviour*"[Title/Abstract] OR "compliant behavior*"[Title/Abstract] OR "compliant behaviour*"[Title/Abstract] OR "teamwork"[Title/Abstract] OR "team work"[Title/Abstract] OR "collective decision*"[Title/Abstract] OR "Referral and Consultation"[MeSH Terms:noexp] OR "referral*"[Title/Abstract] OR "consultation*"[Title/Abstract] OR "Interprofessional Relations"[MeSH Terms] OR "interprofessional relation*"[Title/Abstract] OR "inter professional relation*"[Title/Abstract] OR "interdisciplinary communication"[Title/Abstract] OR "inter-disciplinary communication"[Title/Abstract] OR "multidisciplinary communication"[Title/Abstract] OR "multi-disciplinary communication"[Title/Abstract] OR "cross disciplinary communication"[Title/Abstract] OR "transdisciplinary communication"[Title/Abstract] OR "interdisciplinary collaboration*"[Title/Abstract] OR "inter-disciplinary collaboration*"[Title/Abstract] OR "multidisciplinary collaboration*"[Title/Abstract] OR "multi-disciplinary collaboration*"[Title/Abstract] OR "cross disciplinary collaboration*"[Title/Abstract] OR "transdisciplinary collaboration*"[Title/Abstract] OR "trans-disciplinary collaboration*"[Title/Abstract]*

FILTER: “last ten years”
